# Trace Level Determination of Polyether Ionophores in Feed

**DOI:** 10.1155/2013/151363

**Published:** 2012-12-04

**Authors:** Mervi Rokka, Marika Jestoi, Kimmo Peltonen

**Affiliations:** ^1^Chemistry and Toxicology Unit, Research and Laboratory Department, Finnish Food Safety Authority Evira, Mustialankatu 3, 00790 Helsinki, Finland; ^2^Product Safety Unit, Control Department, Finnish Food Safety Authority Evira, Mustialankatu 3, 00790 Helsinki, Finland

## Abstract

A liquid chromatography-mass spectrometric method was developed and validated to determine six polyether ionophores (lasalocid sodium, monensin sodium, salinomycin sodium, narasin, maduramicin ammonium alpha, and semduramicin sodium) in feed samples. The method developed was very straightforward, involving extraction with 84% acetonitrile of the coccidiostats from the feed samples and filtration of the raw extract prior to chromatographic analysis. Method validation included the determination of selectivity, linearity, specificity, repeatability, the limit of detection, limit of quantification, decision limit (CC**α**), detection capability (CC**β**), and recovery. Feed samples from the Finnish national feed control programme and suspected carry-over samples from a feed manufacturer were analysed in parallel with an existing liquid chromatography method coupled with ultraviolet detection. All feed control samples were negative in LC-UV, but with the developed MS method, monensin, salinomycin, and narasin were detected at concentrations of <0.025–0.73 mg/kg, <0.025–0.027 mg/kg, and <0.025–1.6 mg/kg, respectively. In suspected carry-over samples after an output of 2.0 tonnes of unmedicated feed in the pelletizer line, the concentrations of monensin, salinomycin, and narasin varied from undetected to 16 mg/kg. In the mixer line, after 3.2 tonnes of unmedicated feed output, the concentrations of monensin, salinomycin, and narasin varied from undetected to 2.4 mg/kg.

## 1. Introduction

**Figure 1:**
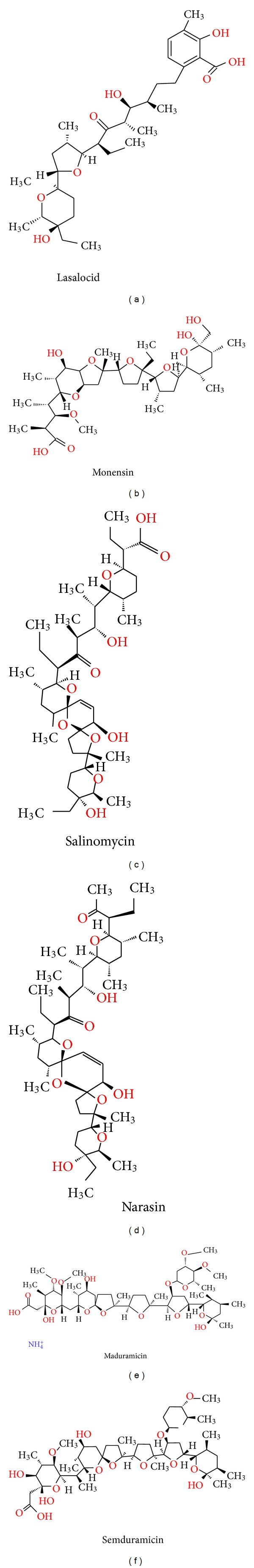
The structures of polyether ionophores.

The polyether ionophores lasalocid, monensin, salinomycin, narasin, maduramicin, and semduramicin (Figure 1) are widely used feed additives in the poultry industry to prevent and control coccidiosis caused by the genus *Eimeria*. They are added to feeds as their sodium salts at levels of 75–125 mg/kg (lasalocid), 60–125 mg/kg (monensin), 20–70 mg/kg (salinomycin), 60–70 mg/kg (narasin), 5 mg/kg (maduramicin), and 20–25 mg/kg (semduramicin). Broiler chickens receive coccidiostats via feed during their entire lifespan, whereas for egg-laying birds the use of coccidiostats is forbidden. Anticoccidial drugs can also be administered, for example, to cattle to improve feed efficacy, that is, to increase their body mass. Due to the extensive use of coccidiostats, the safety of these compounds has been tested in many animal experiments (in both target and nontarget animal species). Although monensin, salinomycin, narasin, and maduramicin are forbidden for laying hens, these compounds were not found to have significant effects, for instance, on egg production or shell thickness when used at the permitted levels [1]. The safe dose levels of coccidiostats for cattle have been noted to be lower than the feeding levels for poultry, for example, monensin 30–45 mg/kg [2], lasalocid 10–35 mg/kg, [3] and narasin 25 mg/kg [4]. Although maduramicin 0–10 mg/kg and semduramicin 0–25 mg/kg were reported to have no effects on horses [5, 6], in a feeding trial on horses with narasin at the level of 80 mg/kg, some reduction in feed intake was observed [4]. Coccidiostats are not used in human medicine because of their cardiovascular effects [7], and indeed lasalocid (<1.0 *μ*M) has been found to cause contraction of the human heart in test systems [8]. However, the residues of coccidiostats in food have not induced any short-term clinical symptoms in humans [9].

A withdrawal period of 5 days is required for lasalocid, salinomycin, narasin, maduramicin, and semduramicin, and 3 days for monensin in order to avoid coccidiostat residues in edible tissues, although there is no risk to consumers' health from ingestion of coccidiostats residues in tissues of animals exposed to feed cross-contaminated up to a level of 10%. [e.g., 4-6] Lasalocid, monensin, and salinomycin are used for laying birds up to the age of 12–16 weeks, depending on the additive. None of the coccidiostats are licensed for the use in egg-laying birds and therefore eggs should be free from coccidiostats contamination. Nonetheless, residues of polyether ionophores have been detected in European eggs at levels from 0.3 to <40 *μ*g/kg [10–14]. In addition, traces of polyether ionophores have found in different European poultry tissues at levels of 0.04–4.2 *μ*g/kg [12, 15, 16]. However, in most samples the concentrations of these compounds have been below the limits of quantification. 

Several methods used to determine polyether ionophores in feed are based on high performance liquid chromatography (HPLC) with fluorescence detection [17, 18] or UV detection [19–23]. In these methods, sample preparation includes precolumn or postcolumn derivatization or solid phase extraction. The limits of quantification (LOQ) in HPLC-based methods are mainly higher than 1 mg/kg, with some exceptions for lasalocid and narasin (LOQ 0.5 mg/kg) [17, 20]. Liquid chromatography-mass spectrometry (LC-MS) was also used to determine polyether ionophores in feed [24–29]. The use of a mass spectrometer detector enables sample preparation to be minimized and also increases the sensitivity of the method in the analysis of complex sample matrices, since it can be used for either selected ion monitoring or multiple reaction monitoring to detect only the desired ions produced by the analytes. However, in these MS methods, apart from the study of Huang et al. [29], solid phase extraction has been used in sample preparation. With the methods mentioned above, polyether ionophores can be detected at levels of 0.001–50 mg/kg. Although the limits of quantification are low (*μ*g/kg), most of the methods have been validated at the mg/kg level. In addition, apart from the study of Delahaut et al. [30], the validated methods have only been tested with medicated feeds, not with real samples from the feed industry. Delahaut et al. [30] analysed feed samples in which the concentrations of monensin, salinomycin, and narasin were above 2.1 mg/kg. The reported findings of polyether ionophores residues in eggs and tissues have been attributed to the contamination of unmedicated feed at the feed manufacturer [11, 14]. For instance, lasalocid is known to be a very dusty compound that can easily contaminate feed during the manufacturing process. For this reason, a suitable method is needed for analysing polyether ionophores at the trace level in feed.

The European Commission has established official maximum contents of polyether ionophores in feed (lasalocid 1.25 mg/kg; narasin 0.7 mg/kg; salinomycin 0.7 mg/kg; monensin 1.25 mg/kg; semduramicin 0.25 mg/kg; maduramicin 0.05 mg/kg) [31]. Based on this regulation, the aims of our study were to (i) develop and validate an LC-MS/MS method for analysing lasalocid, monensin, salinomycin, narasin, maduramicin, and semduramicin at the required performance levels in feed; (ii) apply the validated method in the analysis of both medicated and contaminated feed; and (iii) compare the results determined with LC-MS/MS and the existing LC-UV method. 

## 2. Materials and Methods 

### 2.1. Samples

A cereal mixture (wheat/rye/barley, 1 : 1 : 1, w/w/w) was used in the validation study. The validated method was used in analysis of poultry feed from the Finnish national feed control programme (2008: 8 samples) and suspected carry-over samples from a European feed manufacturer (2006: 12 samples; 2008: 12 samples). The carry-over of monensin, salinomycin, and narasin from medicated feed to unmedicated feed was investigated. Three batches of unmedicated feed were collected after one batch of medicated feed. Unmedicated feed samples from the mixer line were collected after 1 min (0.8 tonnes output), 2.5 min (2.0 tonnes), and 4 min (3.2 tonnes). In proportion, unmedicated feed samples from the pelletizer line were collected after 3 min (0.6 tonnes output), 5 min (1.0 tonnes), and 10 min (2.0 tonnes). 

### 2.2. Chemicals and Reagents

Standards of monensin (sodium salt, ~90–95%), salinomycin (~96%), and narasin (~97%) were purchased from Sigma (St. Louis, MO, USA), lasalocid (sodium salt) from Fluka Chemie (Buchs, Switzerland), maduramicin (ammonium, ~96.5%) from Alpharma (Willow Island, NE, USA), and semduramicin from BVL (Braunschweig, Germany). Standard stock solutions (1 mg/mL) and working standard solutions for LC-MS/MS analysis (monensin, salinomycin, narasin, maduramicin: 1 *μ*g/mL & 10 *μ*g/mL; lasalocid, semduramicin; 8 *μ*g/mL & 80 *μ*g/mL) were prepared in methanol. Standard stock solutions for HPLC (0.5 mg/mL) were prepared in methanol. Working standard solutions for HPLC were prepared in methanol : water (90 : 10, v/v). Methanol, acetonitrile, acetic acid (analytical grade), ammonium acetate, and sulphuric acid were purchased from J. T. Baker (Deventer, Holland). Vanillin (99%) was purchased from Fluka Chemie (Buchs, Switzerland). A vanillin postcolumn reagent was freshly prepared daily by dissolving 30 g vanillin in 970 mL methanol/sulphuric acid (20 mL sulphuric acid added to 950 mL methanol, with the solution then cooled in an ice bath). The water used was purified with a Millipore Milli-Q Plus System (Millipore, Espoo, Finland). 

### 2.3. LC-MS/MS: Sample Preparation and Analysis

Samples were ground with a laboratory mill (Bamix, Mettlen, Switzerland). Then, 25 g of ground sample was extracted with 100 mL of 84% acetonitrile in water for two hours using a VKS-75 horizontal shaker (Edmund Bühler, Bodelshansen, Germany) at room temperature. Samples in the validation study were spiked with different concentrations of polyether ionophores standard solutions just before extraction. As the matrix of carry-over samples was different than matrix used in the validation, the samples were also spiked before extraction to ensure suitability of the method for these samples. The extracted samples were filtered through S&S 602 H 1/2 filter paper (Schleicher & Schuell, Dassal, Germany), and the filtered extracts were stored at +4°C until analysis. One millilitre of extract was evaporated to dryness under nitrogen at 40°C. The sample was dissolved in 1 mL of mobile phase (acetonitrile: 2 mM ammonium acetate containing 2% acetic acid (95 : 5 v/v)) and filtered using a 0.2-*μ*m syringe filter (Pall Gelamn Sciences, Ann Arbor, MI, USA) into an autosampler vial. 

Polyether ionophores were analysed with a Waters Alliance 2695 liquid chromatograph (Waters, Milford, MA, USA) connected to a MicroMass Quattro Micro triple-quadrupole mass spectrometer (Micromass, Manchester, UK). The separation of polyether ionophores was conducted as described by Jestoi et al. [16]. The analytical column was a Luna C_18_(2) (5 *μ*m), 3.0 × 150 mm (Phenomenex, Cheshire, UK). The flow rate of the mobile phase was 0.5 mL/min and the injection volume was 10 *μ*L. A positive ionisation mode was used with an ESI probe. The parameters of the MS were optimized using a standard solution. The best response was recorded with the following parameters: capillary voltage 3.75 kV, source temperature 130°C and desolvation temperature 250°C. The optimized cone voltages and collision gas energies for each of the analysed coccidiostats are presented in Table 1. Argon (AGA, Finland) was used as a collision gas. Coccidiostats were detected as either their sodium or potassium adducts. 

### 2.4. LC-UV: Sample Preparation and Analysis

The LC-UV method for polyether ionophores was modified from the method AM-AA-CR-J424-AC-791 [32]. Briefly, 5 g of ground sample was extracted with 50 mL of 90% methanol in water for one hour at room temperature. After extraction, 50 mL of extraction solution was added and sample solids were allowed to settle. The sample solution was filtered using a 0.45 *μ*m syringe filter (Pall Gelamn Sciences, Ann Arbor, MI, USA) into an autosampler vial.

Monensin, salinomycin, and narasin were analysed using an Agilent 1100 liquid chromatograph (Agilent, Waldbron, Germany) and postcolumn reagent pump (Shimadzu Instruments, Maryland, USA). Coccidiostats were detected at 520 nm. The analytical column was a Shandon 250 × 4.6 mm Hypersil ODS (5 *μ*m). The flow rate was 0.8 mL/min for the mobile phase (methanol : water : acetic acid, 94 : 6 : 1, v/v/v) and 0.7 mL/min for the vanillin reagent. The injection volume was 50 *μ*L. In this method, the limit of detection (LOD) for monensin, salinomycin, and narasin was 2 mg/kg, 2 mg/kg, and 3 mg/kg, respectively, and the respective limits of quantification (LOQ) for these analytes were 4 mg/kg, 4 mg/kg, and 5 mg/kg.

### 2.5. Validation of the LC-MS/MS Method

Method validation included the determination of selectivity, linearity, specificity, reproducibility, recovery, the decision limit (CC*α*), detection capability (CC*β*), limit of detection (LOD), and limit of quantification (LOQ). Six replicates of samples spiked at three concentrations (spiking level 1: monensin, salinomycin, narasin, and maduramicin 0.025 mg/kg, lasalocid and semduramicin 0.20 mg/kg; spiking level 2: monensin, salinomycin, narasin, and maduramicin 0.05 mg/kg, lasalocid and semduramicin 0.40 mg/kg; spiking level 3: monensin, salinomycin, narasin, and maduramicin 0.10 mg/kg, lasalocid and semduramicin 0.80 mg/kg) and calibration curves with and without the matrix were analysed on three separate days. The calibration curves were constructed with external standards by injecting standards prepared in the mobile phase and matrix at five different concentrations (monensin, salinomycin, narasin, and maduramicin 0.010–2.0 mg/kg; lasalocid and semduramicin 0.080–2.0 mg/kg). The blank matrix was spiked with corresponding amounts of standards and prepared as regular samples.

## 3. Results

### 3.1. LC-MS/MS Method Development

The LC-MS/MS method was developed to provide confirmatory data for the analysis of six polyether ionophores in feed. Sample preparation was simple, involving the extraction of coccidiostats from feed samples and filtration of the extract prior to chromatographic analysis. As the chemical structures and particularly the mode of action of coccidiostats and emerging *Fusarium*-mycotoxins are closely related, we utilized earlier published sample extraction method for beauvericin and enniatins [33]. The MS/MS fragmentation conditions and collision energies were optimised for each individual compound to give the best response. The compounds were separated with an earlier-developed LC method for separating lasalocid, monensin, salinomycin, narasin, and maduramicin in eggs and tissues [16]. In this method development, the sixth compound, semduramicin could be added as such to the already existing LC method.

### 3.2. LC-MS/MS Method Validation

**Figure 2:**
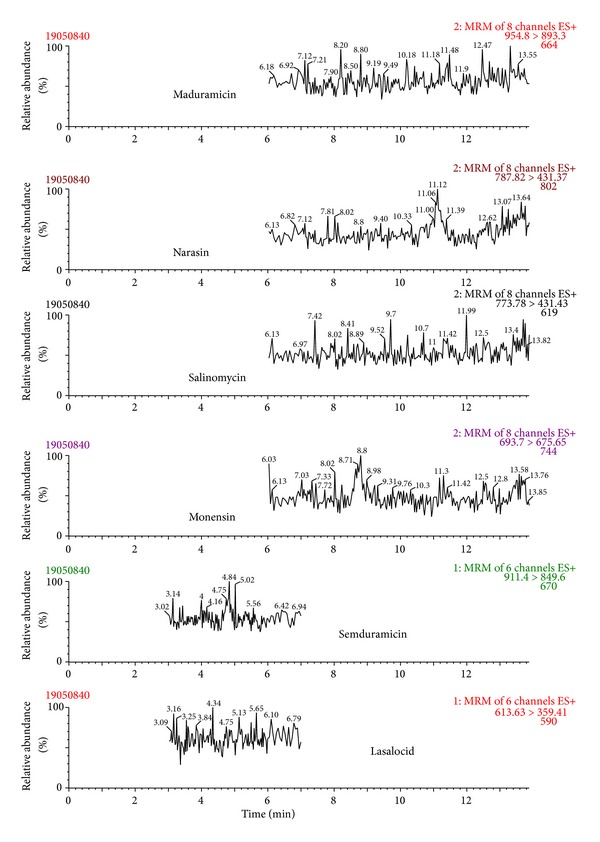
LC-MS/MS chromatogram of a blank feed sample.

**Figure 3:**
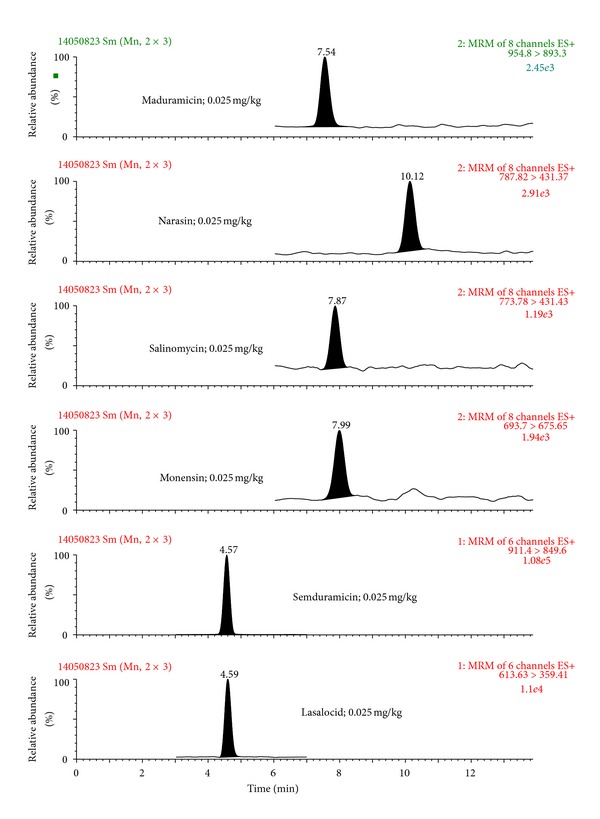
LC-MS/MS chromatogram of a spiked feed sample at the levels of 0.025 for monensin, salinomycin, narasin, and maduramicin and 0.20 mg/kg for lasalocid and semduramicin.

**Figure 4:**
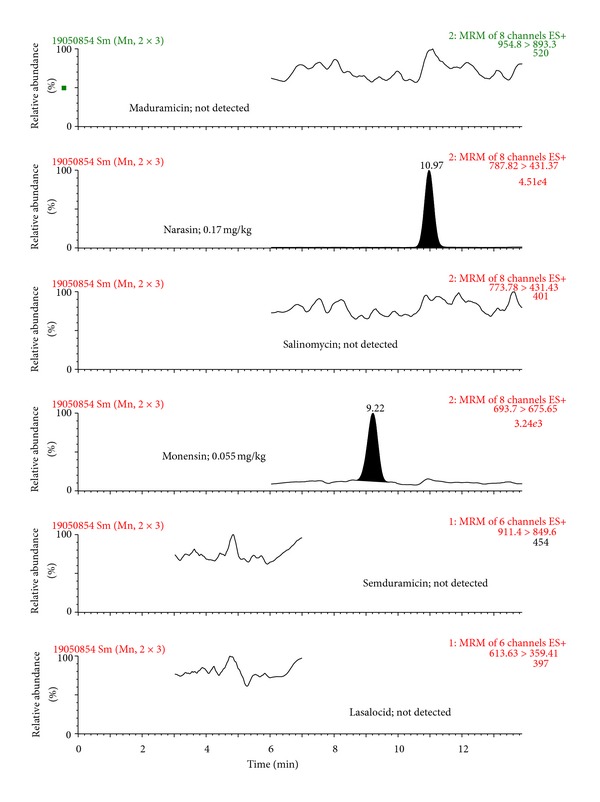
LC-MS/MS chromatogram of a positive feed sample; monensin at the level of 0.055 mg/kg, narasin at the level of 0.17 mg/kg.

The selectivity of the developed method was tested by comparing (two-sided *t*-test) the slopes of the five-point calibration curves obtained with and without the matrix (for calibration curve ranges, see section Validation of the LC-MS/MS method). Due to the presence of significant matrix effects (*P* < 0.05), the calibration curves for all compounds were prepared in the matrix. The matrix effect, calculated as the ratio of signal suppression to enhancement (SSE%) as described in Sulyok et al. [34], was 3.0%, 4.1%, 2.8%, 4.2%, 19%, and 43% for lasalocid, monensin, salinomycin, narasin, maduramicin, and semduramicin, respectively. This further demonstrated the suppressive effect of the matrix components on the signal intensity. The acceptability of linearity of each point of the matrix-assisted calibration curves was tested by using the least-square method [35]. The maximum response/mass ratio of ±10% for each calibration point was accepted. On this basis, it can be stated that the measurement of polyether ionophores in the matrix was linear over the actual studied ranges (0.010–2.0 mg/kg). The specificity of the method was tested using by analysing 20 different blank samples separately and no interference signals close to the retention times of polyether ionophores were detected in any blank samples analysed. Chromatograms of a blank sample, a spiked sample, and a positive sample are presented in Figures 2, 3, and 4, respectively. 

LOD and LOQ values for polyether ionophores were calculated using the responses of blank samples (*n* = 20). LODs (blank sample mean response + 3 × standard deviation) for lasalocid, monensin, salinomycin, narasin, maduramicin, and semduramicin were 0.0029 mg/kg, 0.0042 mg/kg, 0.0047 mg/kg, 0.0026 mg/kg, 0.0011 mg/kg, and 0.0022 mg/kg, respectively. The corresponding LOQs (blank sample mean response + 10 × standard deviation) for the compounds analysed were 0.0075 mg/kg, 0.011 mg/kg, 0.012 mg/kg, 0.0071 mg/kg, 0.0029 mg/kg, and 0.0060 mg/kg, respectively. Although lower concentrations of the analytes could be quantified with the method, for practical reasons the LOQs used for the analytes were the lowest spiking level of 0.025 mg/kg for monensin, salinomycin, narasin, and maduramicin, and 0.080 mg/kg for lasalocid and semduramicin. CC*α* and CC*β* values were calculated using data on within-laboratory reproducibility from this study (data not shown). CC*α* and CC*β* values for monensin, salinomycin, narasin, and maduramicin were 0.025–0.027 mg/kg and 0.029–0.033 mg/kg, respectively. CC*α* values for lasalocid and semduramicin were 0.37 mg/kg and 0.24 mg/kg, respectively, and the corresponding CC*β* values, in turn, were 0.50 mg/kg and 0.29 mg/kg (Table 2). 

The mean recoveries of six replicates on three separate days at three different spiking levels of the analytes are presented in Table 3. The recoveries of coccidiostats in feed varied between 74–112%, which was acceptable for monitoring purposes. In addition, the method was repeatable, as demonstrated by the relative standard deviations of the mean recoveries (Table 3).

### 3.3. Sample Analysis with LC-MS/MS

To ensure the usefulness of the LC-MS/MS method for the detection of polyether ionophores, especially at trace levels, feed samples from the Finnish national feed control programme and suspected carry-over samples from a European feed manufacturer were analysed with the developed method. For the analyses of medicated feed there was a need for a wider calibration curve (10–100 mg/kg), which was also tested to be linear over the range. For that reason, carry-over samples could be analysed with any dilution. The carry-over samples were also spiked and the result was corrected by recovery, if the recovery % was over the range recoveries obtained during the validation. Lasalocid, maduramicin, and semduramicin were not detected in any samples analysed with LC-MS/MS. In samples from the feed control programme the concentrations of monensin and narasin were <0.025–0.73 mg/kg and <0.025–1.6 mg/kg, respectively (Table 4). Salinomycin was not detected in any feed control samples. 

In the suspected carry-over samples from the pelletizer line, after an output of 2.0 tonnes of unmedicated feed, the level of monensin was 8 mg/kg and 16 mg/kg in two turkey feeds. In two broiler feeds, the concentration of narasin after 2.0 tonnes of unmedicated feed output was 16 mg/kg and 17 mg/kg. Carry-over was also detected in the mixer line, but it was minor compared to the pelletizer line. After 3.2 tonnes of unmedicated feed output, the concentration of monensin in feed for turkey and broiler was 0.58–2.4 mg/kg and <0.025–0.27 mg/kg, respectively. The concentration of narasin was 0.16–0.28 mg/kg in turkey feed and 0.094–1.5 mg/kg in broiler feed. Salinomycin was detected in nine samples (<0.025–0.027 mg/kg) from both the pelletizer and mixer lines. All the results for these suspected carry-over samples are presented in Table 5.

### 3.4. Sample Analysis with LC-UV

The samples mentioned in Section 3\tmspace +\thinmuskip {.1667em}3.3 were also analysed for their content of monensin, salinomycin, and narasin with the already existing LC-UV method to compare the results with those determined with LC-MS/MS. The samples from the feed control programme were all negative in LC-UV. In carry-over samples, salinomycin was also not found using LC-UV. The concentration of monensin in six feed samples for turkey varied between 6.9 mg/kg and 36 mg/kg, which was slightly lower than determined using LC-MS/MS (8–39 mg/kg). In addition, the content of narasin in six broiler feed samples was lower when analysed with LC-UV (8.3–39 mg/kg) than with LC-MS/MS (16–70 mg/kg). In other carry-over samples, the concentrations of monensin and narasin ranged from undetected to <5 mg/kg when analysed with LC-UV (Table 5).

## 4. Discussion 

The developed LC-MS/MS method proved to be sensitive and repeatable in the analysis of six polyether ionophores, as shown in the validation results. The obtained LOD, LOQ, CC*α*, and CC*β* values demonstrated that the method is capable of quantifying polyether ionophores in feed below the maximum concentrations stated in Commission Directive 2009/8/EC [30]. 

The European Union has not set any recommendation concerning the performance of analytical methods or the interpretation of results for contaminants in feed. Accordingly, the developed LC-MS/MS method was validated according to Commission Decision 2002/657/EC [36], which has been set for residues in food of animal origin. It sets criteria, for example, for the accuracy and precision of quantitative methods. As no CRM (certified reference material) sample was available for polyether ionophores at the trace level, the accuracy of the LC-MS/MS method was determined through the recovery of known amounts of the analytes added to a blank matrix. Although the recovery was under 80% for monensin, salinomycin, maduramicin, and semduramicin at the lowest spiking level, the recoveries were generally repeatable between different days and the method was therefore considered acceptable for analysing polyether ionophores in feed at low levels. The precision of the method was determined from intralaboratory reproducibility. According to Commission Decision 2002/657/EC [36], the coefficient of variation (CV) of intralaboratory reproducibility for concentrations lower 0.10 mg/kg should be as low as possible. The precision of the LC-MS/MS method was in accordance with the criterion. For concentrations over 0.10 mg/kg, the intralaboratory CV would typically be between 12% and 15%. In a few cases this was not met, but overall the method can be considered reproducible for analysing polyether ionophores.

As with other ionophoric compounds, coccidiostats can also form electrically neutral pseudo-macrocyclic complexes with several metal cations, such as Na^+^ and K^+^. Complex formation occurs by intramolecular hydrogen bonding between a carboxylic group at one end of the molecule and a terminal alcohol group at the other [37]. Polyether ionophores are usually detected as their sodium adducts in tissues and eggs samples [38–40]. Sodium adducts of polyether ionophores have also been detected in feed [26, 27]. In this study, during method testing, both sodium and potassium adducts were found. Without any control of the MS parameters, the occurrences of these adducts were variable, and thus there were problems in quantification. By optimizing the cone voltages for each of the analysed coccidiostats, the potassium adducts of semduramicin and maduramicin gave a more intensive response than their sodium adducts and were used for both detection and quantification. 

The most widely used method to compensate for matrix effects in LC-MS methods is the use of an internal standard, since this allows the response of an analyte of interest to be normalized, for example, compensating for possible variations during sample preparation, injection, chromatography, and matrix effects. The best choice for an internal standard would be the isotope-labelled analogues of coccidiostats, which would have identical chemical and structural properties to those of the analyte [41]. We observed remarkable increase in the intensity of the compounds when proceeding the sequence. As no internal standards are available for coccidiostats, to solve the problem of increasing intensity, the matrix-assisted calibration curve was run two times before the samples were analysed in order to stabilize the instrument. Moreover, if large numbers of samples were to be analysed, the calibration curve was run after every ten samples and was used to quantify the samples run after the curve, although the increasing of intensity reached a plateau during the analysing run.

**Figure 5:**
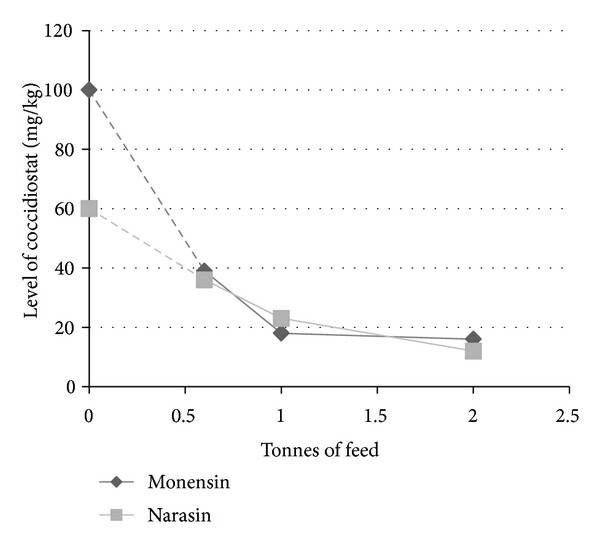
Carry-over of monensin and narasin in the pelletizer line during feed manufacturing.

As the operator control samples from the feed mill demonstrated, cross-contamination is possible during manufacture. Unfortunately, the levels of monensin and narasin in medicated feed were not available. According to the legislation, monensin can be added at concentrations of between 100 and 125 mg/kg to feed for broilers and between 60 and 100 mg/kg to feed for turkeys. Narasin can be added at concentrations of between 60 and 70 mg/kg to feed for broilers. Based on these addition levels, 10–20% of the added monensin was still left after three unmedicated feed batches (2.0 tonnes) in the pelletizer line (Figure 5). In addition, about 20% of the added narasin was left in the same pelletizer line. When unmedicated feed was run in the mixer line, about 2% of the added levels of monensin and narasin were left after three batches. When comparing the concentrations of monensin and narasin in unmedicated feed with the maximum content, the concentrations in feed were much higher than the permitted levels. As the analysed feeds were not fed to poultry, no data were available on the transfer of these contaminants to eggs or tissues. Kennedy et al. [11] reported the carry-over of lasalocid from medicated feed to unmedicated batches of feed during manufacture. Lasalocid was found at levels as high as 0.5–1.0 mg/kg in the ninth batch of unmedicated feed, a level high enough to result in residues in eggs.

The results determined with the developed LC-MS/MS method and the existing LC-UV method were compared. In those carry-over samples in which the concentrations of coccidiostats were more than 10 mg/kg, the results determined with both methods were similar. As expected, significant differences between the results of two analytical methods were observed at trace levels of coccidiostats, since the LOQ values of the LC-MS/MS method (0.025–0.080 mg/kg) were much lower than those of the LC-UV method (4-5 mg/kg). Samples from the national feed control programme were collected from feed batches that should not contain any polyether ionophores. With the LC-UV method, samples were previously found to be negative for monensin, salinomycin, and narasin. However, with the LC-MS/MS method, both monensin and narasin were detected at concentrations ranging from <0.025–0.73 mg/kg and <0.025–1.6 mg/kg, respectively. It is notable that narasin was found in one sample at the concentration of 1.6 mg/kg, which exceeded the permissible content (0.7 mg/kg). Moreover, in those carry-over samples that were found to be negative for monensin and narasin with the LC-UV method, both compounds were detected with the LC-MS/MS method. Thus, the developed LC-MS/MS method proved to be suitable for detecting trace amounts of polyether ionophores in feed.

## 5. Conclusion

In conclusion, a new LC-MS/MS method was developed for the determination of six polyether ionophores at trace levels. Sample preparation was minimized, because the raw extracts were only filtered before the LC run. The use of mass spectrometry enabled polyether ionophores to be analysed without precolumn or postcolumn derivatization. The validation data indicate that the developed method is reliable in the determination of coccidiostats in feed at the existing contamination levels. The LC-MS/MS method demonstrated its efficiency in the analysis of coccidiostats at trace levels against the conventional LC method. In feed samples that tested negative with the LC-UV method, concentrations of coccidiostats of up to 2.10 mg/kg were detected with LC-MS/MS. The suspected carry-over of coccidiostats during the manufacture of feed was tested by analysing unmedicated feed batches produced after medicated feed. Coccidiostats could be still detected after 3.2 tonnes of unmedicated feed output.

## Figures and Tables

**Table 1:** LC-MS/MS acquisition for polyether ionophores. The product ions (in bold) were used for the quantification.

Compound	Precursor ion (m/z)	Product ions	Cone Voltage (kV)	Collision gas energy (eV)
Lasalocid	613.6 [M + Na]^+^	359.4; **377.4**	50	40
Semduramicin	911.4 [M + K]^+^	**849.6**; 867.0	34	40
Monensin	693.7 [M + Na]^+^	461.4; **675.7**	60	40
Salinomycin	773.8 [M + Na]^+^	**431.4**; 531.5	70	45
Narasin	787.8 [M + Na]^+^	**431.4**; 531.5	60	50
Maduramicin	954.8 [M + K]^+^	**893.3**; 911.3	34	40

**Table 2:** CC*α* (mg/kg) and CC*β* (mg/kg) values obtained for polyether ionophores in feed.

	Lasalocid	Monensin	Salinomycin	Narasin	Maduramicin	Semduramicin
CC*α* (mg/kg)	0.37	0.025	0.025	0.027	0.027	0.24
CC*β* (mg/kg)	0.50	0.030	0.029	0.031	0.033	0.29

**Table 3:** The mean recoveries with the corresponding standard deviations of the polyether ionophores analysed at three spiking levels (*n* = 18).

	Spiking level (mg/kg)	Mean concentration (mg/kg)	Recovery %	Relative standard deviation (%)
Lasalocid	0.20	0.19	95	35
	0.40	0.45	112	5.0
	0.80	0.76	95	6.0

Monensin	0.025	0.019	75	13
	0.05	0.050	100	26
	0.10	0.10	100	9.2

Salinomycin	0.025	0.19	77	13
	0.05	0.054	108	12
	0.10	0.097	97	9.8

Narasin	0.025	0.020	81	112
	0.05	0.054	109	9.5
	0.10	0.096	96	5.9

Maduramicin	0.025	0.019	74	18
	0.05	0.055	109	9.5
	0.10	0.095	95	7.4

Semduramicin	0.20	0.15	77	20
	0.40	0.43	106	17
	0.80	0.74	92	16

**Table 4:** Samples from the Finnish national feed control programme from 2008 analysed by LC-MS/MS.

	Monensin (mg/kg)	Narasin (mg/kg)
Feed 1	0.046	<0.025
Feed 2	0.73	<0.025
Feed 3	<0.025	0.26
Feed 4	0.059	0.47
Feed 5	<0.025	0.12
Feed 6	0.080	1.6
Feed 7	0.036	0.26
Feed 8	0.055	0.17

Lasalocid, maduramicin, and semduramicin were not detected with LC-MS/MS. All samples analysed with HPLC-UV were negative. Lasalocid, maduramicin, and semduramicin were not measured with HPLC-UV.

**Table 5:** Levels (mg/kg) of polyether ionophores in carry-over samples (turkey and broiler feed) from pelletizer and mixer lines analysed by both LC-MSMS and LC-UV.

Feed	Tonnes of feed	Monensin		Salinomycin		Narasin	
		LC-MSMS	LC-UV	LC-MSMS	LC-UV	LC-MSMS	LC-UV
Turkey A	0.6	29	17	<0.025	n.d.	1.9	n.d.
pelletizer	1.0	17	11	<0.025	n.d.	1.2	n.d.
line	2.0	8.0	6.9	<0.025	n.d.	0.74	n.d.

Turkey B	0.6	39	36	n.d.	n.d.	1.3	n.d.
pelletizer	1.0	18	16	n.d.	n.d.	0.60	n.d.
line	2.0	16	16	n.d.	n.d.	0.52	n.d.

Turkey C	0.8	0.60	n.d.	n.d.	n.d.	0.16	n.d.
mixer	2.0	0.73	n.d.	n.d.	n.d.	0.14	n.d.
line	3.2	0.58	n.d.	n.d.	n.d.	0.16	n.d.

Turkey D	0.8	5.3	4.3	n.d.	n.d.	0.35	n.d.
mixer	2.0	6.0	<4.0	n.d.	n.d.	0.24	n.d.
line	3.2	2.4	<4.0	n.d.	n.d.	0.28	n.d.

Broiler A	0.6	3.6	<4.0	n.d.	n.d.	70	39
pelletizer	1.0	3.6	4.3	n.d.	n.d.	51	32
line	2.0	3.3	4.1	n.d.	n.d.	16	14

Broiler B	0.6	0.070	n.d.	0.027	n.d.	47	30
pelletizer	1.0	0.066	n.d.	<0.025	n.d.	33	20
line	2.0	0.031	n.d.	<0.025	n.d.	17	8.3

Broiler C	0.8	<0.025	n.d.	<0.025	n.d.	6.0	4.0
mixer	2.0	<0.025	n.d.	<0.025	n.d.	0.54	n.d.
line	3.2	<0.025	n.d.	<0.025	n.d.	0.094	n.d.

Broiler D	0.8	0.35	n.d.	n.d.	n.d.	1.9	<5.0
mixer	2.0	0.33	<4.0	n.d.	n.d.	2.1	n.d.
line	3.2	0.27	n.d.	n.d.	n.d.	1.5	n.d.

n.d.: not detected

Lasalocid, maduramicin, and semduramicin were not detected with LC-MS/MS. Lasalocid, maduramicin, and semduramicin were not measured with LC-UV.
